# Type‐Specific Single‐Neuron Analysis Reveals Mitochondrial DNA Maintenance Failure Affecting Atrophying Pontine Neurons Differentially in Lewy Body Dementia Syndromes

**DOI:** 10.1111/acel.70125

**Published:** 2025-06-06

**Authors:** Eloise J. Stephenson, Laura J. Bailey, Stephen Gentleman, Helen Tuppen, Istvan Bodi, Claire Troakes, Christopher M. Morris, Tony M. Carr, Sarah Guthrie, Joanna L. Elson, Ilse S. Pienaar

**Affiliations:** ^1^ Department of Neuroscience, School of Life Sciences University of Sussex Brighton UK; ^2^ Genome Damage and Stability Centre, School of Life Sciences University of Sussex Brighton UK; ^3^ Department of Brain Sciences Imperial College London London UK; ^4^ Centre for Mitochondrial Research, The Biosciences Institute Newcastle University Newcastle upon Tyne UK; ^5^ London Neurodegenerative Diseases Brain Bank, Institute of Psychiatry, Psychology and Neuroscience King's College London London UK; ^6^ Newcastle Brain Tissue Resource Newcastle University Newcastle upon Tyne UK; ^7^ Centre for Human Metabolomics North‐West University Potchefstroom South Africa; ^8^ Institute of Clinical Sciences, School of Biomedical Sciences University of Birmingham Birmingham UK

**Keywords:** brainstem, cholinergic neurons, Lewy body dementia, mitochondrial DNA, noradrenergic neurons, nuclear gene transcriptomic responses

## Abstract

The age‐associated neurodegenerative disorder, Lewy body dementia (LBD), encompasses neuropsychiatric symptom‐overlapping Dementia with Lewy bodies (DLB) and Parkinson's Disease with Dementia (PDD). We characterised how differential mitochondrial DNA (mtDNA) profiles contribute to neurotype‐specific neurodegeneration and thereby clinicopathological heterogeneity, between LBD's syndromes. We further characterised key nuclear‐encoding genes' recalibrations in response to such mtDNA changes. In post‐mortem ‘single‐cell’ acetylcholine‐ and noradrenaline‐producing neurons, respectively of the pedunculopontine nucleus (PPN) and locus coeruleus (LC) from DLB, PDD and neurological‐control brains, we quantified ‘major arc’‐locating mtDNA deletions (mtDels) and ‐copy number (mtCN), and measured mRNA levels of nuclear‐encoding genes regulating mtDNA maintenance, ‐biogenesis and mitophagy. DLB cases' OXPHOS defect instigating mtDel burden was higher in both neurotypes than PDD. In DLB, mtCN was reduced for both neurotypes, but PDD cases revealed mtDNA depletion in LC‐noradrenergic neurons only. DLB patients' shorter survival correlated with PPN‐cholinergic neurons' mtDel levels, inversely with wild‐type mtCN, implying that such neurons' inability to maintain sufficient wild‐type mtDNA content drive DLBs' rapid psycho‐cognitive manifestations. Contrastingly, PDD's longer disease duration allowed compensation against mtDels' clonal expansion in PPN‐cholinergic neurons. Moreover, PDD induced mRNA depletion of a mitochondrial genome maintenance gene in PPN‐cholinergic neurons, whilst LC‐noradrenergic neurons displayed reduced expression of a mitophagy regulating gene. Here we identify mitochondrial genome maintenance and mitophagy pathway enrichment as therapeutic targets to offset defective mtDNA within pontine cholinergic and noradrenergic neurons of PDD patients. The pronounced LBD subtype‐related mitochondria‐nuclear genetic differences question the consensus that pathology converges at disease end‐stage, calling for LBD subtype and neurotype‐specific therapeutics.

## Graphical abstract

(a) Mitochondria contain wild‐type (black)/mutant (red) mtDNA; exceeding the mtDNA mutation threshold compromises OXPHOS, potentially driving degeneration of neurons vulnerable to age‐related neurodegenerative disease. Cellular compensations that is, mtDNA copy number upregulation and crosstalk with nuclear DNA may fail, driving neurodegeneration further. (b) We recorded levels of deletions affecting the mtDNA's ‘major arc’, mtDNA copy number and nuclear DNA gene responses in neurodegenerating pontine neurotypes, revealing defective maintenance of a healthy mtDNA pool as an important disease mechanism in both LBD phenotypes, but neurotype‐dependent spectrum opposing mitochondrial homeostatic profiles.

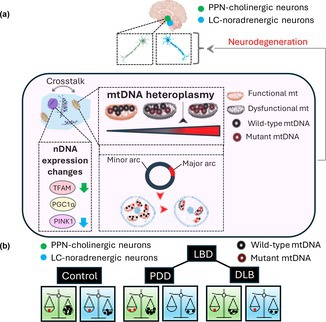

**Abbreviations**
AD: Alzheimer's disease
Aβ: β‐amyloid
CERAD: Consortium to Establish a Registry for Alzheimer's Disease
CRISPR: Clustered Regularly Interspaced Short Palindromic Repeats
DLB: Dementia with Lewy bodies
DoD: Duration of disease
ETC: Electron transport chain
LC: locus coeruleus
LQ: lower quartile
MDS: mtDNA depletion syndrome
mtCN: mtDNA copy number
mtDels: mtDNA deletions
mtDNA: Mitochondrial DNA
nDNA: nuclear DNA
PD: Parkinson's disease
PDD: Parkinson's Disease Dementia
PGC: peroxisome proliferator‐activated receptor‐gamma coactivator
PINK: phosphatase and tensin homologue‐induced putative kinase
PPN: pedunculopontine nucleus
qPCR: quantitative polymerase chain reaction
RT: room temperature
SEM: standard error mean
TFAM: mitochondrial transcription factor A
TMB: tetramethylbenzidine
UQ: upper quartile
WT: wild‐type

## 1 Introduction

The age‐related disorder, Lewy body dementia (LBD), encompasses two syndromes, namely Parkinson's Disease Dementia (PDD) and Dementia with Lewy bodies (DLB). Neuropathological features for both PDD and DLB include the progressive loss of distinct neurotypes and intracellular proteinaceous aggregates (Lippa et al. 2007), whilst both patient cohorts manifest neuropsychiatric, cognitive and motor symptoms (Emre et al. 2007). However, DLB patients display greater attentional impairment (Downes et al. 1999; Aarsland et al. 2003; Bonanni et al. 2008) and more frequent hallucinations and delusions (Emre et al. 2007; Aarsland et al. 2001, 2005; Mosimann et al. 2006), but milder motor deficits than PDD patients (Molloy et al. 2005).

An international consensus recommends that DLB patients display psycho‐cognitive impairment prior to motor‐related Parkinsonism, whereas PDD patients manifest Parkinsonism before psycho‐cognitive impairment (Emre et al. 2007; American Psychiatric Association 2022; World Health Organization 2019). Increasing recognition of the overlapping yet distinct phenotypes supports the view that these conditions represent points on a spectrum, thereby challenging the validity of this diagnostic rule and implicating different underlying neuropathological substrates underlying PDD versus DLB. Cholinergic and noradrenergic neurons residing, respectively, within synaptically interacting pontine nuclei, namely the pedunculopontine nucleus (PPN) and locus coeruleus (LC), degenerate during LBD (Schmeichel et al. 2008; Brunnström et al. 2011; Del Tredici and Braak 2013; Tilley et al. 2021). The PPN regulates locomotion, arousal, memory and learning (Pienaar et al. 2017). PPN‐deep brain stimulation trials for alleviating gait‐freezing, falls and cognitive dysfunction (Alessandro et al. 2010; Thevathasan et al. 2018) revealed inconsistent results, calling for a more refined targeting strategy, such as exclusive targeting of PPN‐cholinergic neurons, for improving therapeutic responses (Pienaar et al. 2015; Sharma et al. 2020). LC‐noradrenergic neurons regulate memory and executive functions that are left disrupted during LBD (Holland et al. 2021), with noradrenergic drug repurposing that may improve LBD‐associated psycho‐cognitive symptoms (David et al. 2022).

Mitochondrial impairment stemming from somatic mitochondrial DNA (mtDNA) variants, promotes neuronal death during neurodegenerative disease (Pyle et al. 2016; Dölle et al. 2016; Bury et al. 2017; Rajkumar et al. 2020). Our previous work highlighted mtDNA profile differences between different Parkinson's disease (PD)‐susceptible neurotypes (Bury et al. 2017). Here, utilising a ‘single‐cell’, neurotype‐specific analysis approach, we explored whether mtDNA damage profiles differ between degenerating PPN‐cholinergic and LC‐noradrenergic neurons, whilst also distinguishing between LBD's syndromes.

Most mitochondrial proteins are encoded by nuclear genes (Ali et al. 2019), with recognition increasing that acquired mtDNA variation (mtDNA deletions [mtDels] and mtDNA copy number [mtCN]) can cause nuclear DNA (nDNA) transcript responses for maintaining mitochondrial homeostasis (Gupta et al. 2023; Larsson et al. 1998; Wu et al. 2019). Hence, in these disease‐susceptible pontine‐based neurotypes of LBD syndromes, we measured mRNA expression levels of key nuclear‐encoded genes that is, *TFAM* (mitochondrial transcription factor‐A), regulating mtDNA repair (Larsson et al. 1998), *PGC1α* (peroxisome ɤ‐coactivator‐1*α*), regulating mitochondrial biogenesis (Baker and Haynes 2011) and *PINK1* (phosphatase/tensin putative kinase‐1), accumulating on defective mitochondria for mitophagy‐aided clearance (Vives‐Bauza et al. 2010). We anticipated observing upregulated transcript responses, serving as a counter mechanism against rising mtDNA damage that risks neuronal survival, this compensation increasing relative to the neuronal pools' mtDNA integrity loss. Contrastingly, unresponsive or downregulated transcripts presenting with severe mtDNA damage and/or mtDNA depletion could signal disrupted distress communication by mitochondria. The latter scenario would highlight these nDNA‐regulating pathways as important mechanisms by which LBD subtype disease processes drive neurotype‐specific degeneration, hence serving as potential therapeutic targets to offset mtDNA damage that threatens neuronal survival in LBD syndromes.

## 2 Methods

### 2.1 Donors

Sussex University's Research Ethics Committee granted study approval. Informed consent was obtained from donors during life or representatives. Newcastle University's and King's College London's Brain Banks provided PPN/LC‐containing frozen/non‐fixed post‐mortem brain tissue blocks. For each brain donation, both an experienced Consultant Geriatric Psychiatrist and Consultant Neuropathologist affiliated with the respective Brain Bank were responsible for reviewing the clinical study record and the neuropathological report of each case, based on which a consensus diagnosis was then reached. DLB and PDD cases were neuropathologically confirmed in accordance with LBD's diagnostic neuropathological consensus criteria (Attems et al. 2021). DLB cases were further distinguished from PDD ones based on the ‘one‐year rule’ clinical guideline relating to the differential time interval between the development of motor and cognitive symptoms. This recommends diagnosing a patient with PDD when dementia develops in the context of well established PD whilst a diagnosis of DLB entails that dementia precedes or coincides within one year of the development of motor symptoms (Emre et al. 2007; McKeith et al. 2017). As primary ‘synucleinopathy’ disorders, PD and LBD are neuropathologically hallmarked by the abnormal cytoplasmic accumulation of α‐synuclein in the brain. This small (140 amino acids in length) protein can pathologically aggregate into β‐sheet‐rich oligomers and fibrils that form predominantly within neuronal cells and their processes, termed Lewy bodies and Lewy neurites, respectively (Spillantini et al. 1997; Serpell et al. 2000). The pathological delineation of PD compared to PDD/DLB rests on the Lewy bodies/‐neurites lesional stage of progression through the brain. Whereas PD patients' post‐mortem brains reveal that such proteinaceous inclusions are restricted to the brainstem and limbic regions, in PDD and DLB patients the Lewy body neuropathology extends to the neocortex (Walker et al. 2019). Hence, following the differential classification based on patients' clinical presentation (with such information that was derived from patients' clinical case notes), the DLB and PDD clinical cohorts were further distinguished as adhering to either a brainstem‐, limbic‐ or neocortical predominant pathological subgroup, as per criteria put forward by McKeith et al. (2017). Such proteinaceous burden subgrouping of cases is based on the semi‐quantitative scoring of brain region‐based aggregated α‐synuclein deposits, the latter that was detected immunohistochemically by the Brain Banks' technical teams.

Post‐mortem brains that displayed significant concomitant Alzheimer's disease (AD) pathology, conforming to published neuropathologic consensus guidelines for diagnosing AD (Hyman et al. 2012; Montine et al. 2012) were excluded from the study. Specifically, the clinicopathological team associated with each respective Brain Bank applied the ‘ABC’ scoring rubric of the National Institute on Ageing—Alzheimer's Association framework (Montine et al. 2012). This scoring matrix combines Thal phasing for amyloid plaque assessment, Braak staging for neurofibrillary tangles and CERAD (Consortium to Establish a Registry for Alzheimer's Disease) scoring for neuritic plaques to evaluate AD‐related neuropathological changes in post‐mortem brain tissue. Using this composite ‘ABC’ score, it was determined that the extent of AD neuropathological change affecting any of the case material didn't exceed an intermediate extent. The neurological control subjects have not been diagnosed with either a neurodegenerative or pre‐neurodegenerative disease condition.

For analysing PPN‐cholinergic neurons, we investigated *n* = 6 DLB, *n* = 5 PDD and *n* = 6 neurological‐control patients' post‐mortem brains. LC‐noradrenergic neurons from *n* = 6 DLB, *n* = 5 PDD and *n* = 6 neurological‐control cases were analysed. Table S1 summarises patients' information. Non‐fixed/frozen tissue was processed by dissecting the right cerebral hemisphere in the coronal plane. PPN‐containing tissue was taken from the border region between the rostral midbrain and upper pons, including the dorsal pons. The PPN's most caudal pole lies adjacent to the LC, inferior to the cerebellum and apposing the IVth ventricle (Pienaar et al. 2013). The LC [Lat.], meaning ‘blue spot’ due to its neuromelanin deposits, was readily recognisable (Mouton et al. 1994). To determine each case's PPN and LC boundaries, the first and last two 15 μm‐thick sections were histological stained with Luxol Fast Blue and Haematoxylin & Eosin, respectively (Bury et al. 2017) (Figure S1).

### 2.2 Single‐Cell Isolation

PPN‐cholinergic and LC‐noradrenergic neurons were identified immunohistochemically. Glass slide‐mounted tissue sections were air‐dried before washing with 0.1% Tween‐containing Tris‐buffered saline, then blocked with 5% normal horse serum (Vector Laboratories, UK) for 30 min. Cholinergic neurons were labelled with a polyclonal‐goat primary antibody (1:150; Millipore, USA) against choline acetyltransferase, catalysing choline's acetylation to acetylcholine, by incubating sections for 2 h at room temperature (RT). For labelling LC‐noradrenergic neurons, sections were incubated with mouse anti‐noradrenaline transporter primary antibody (1:150; Thermo‐Fisher, USA). Secondary antibodies were horseradish peroxidase‐conjugated horse anti‐goat (1:200; Vector) and horse anti‐mouse secondary antibodies (1:200; Vector) for PPN‐cholinergic and LC‐noradrenergic neurons, respectively. Sections were RT incubated with blue‐staining 3,3′,5,5′‐tetramethylbenzidine (TMB) (Thermo‐Fisher) for 10 min. A UnipicK vacuum‐based cell acquisition system (NeuroInDx, USA), coupled to an inverted microscope (Carl Zeiss, Germany) microdissected individual neurons (Figure S2) (Bailey et al. 2021). Neurons, collected in pre‐autoclaved 1.5 mL Eppendorf tubes, were stored at −20°C until processing. In total, *n* = 722 PPN‐cholinergic neurons were isolated from the DLB, PDD and neurological‐control cases' post‐mortem brains. Of these, *n* = 303 (neurological‐controls: *n* = 107; DLB: *n* = 94; PDD: *n* = 102) were subjected to the mtDel/mtCN measurement assay, resulting in the following mean ± standard error mean (SEM) single neuron sample numbers for each cohort: Neurological‐controls: 16.5 ± 0.61; DLB: 14.3 ± 0.25; PDD: 18.8 ± 0.52. The remaining (*n* = 419) PPN‐cholinergic neurons, as were attained from neurological‐controls (*n* = 122; 9.8 ± 0.2 [mean ± SEM]), DLB (*n* = 160; 10.4 ± 0.2) and PDD (*n* = 137; 10.7 ± 0.3) post‐mortem brains, were utilised for measuring *mRNA* expression levels of the target nuclear‐encoded genes. For LC‐noradrenergic neuronal analysis, a total of *n* = 537 single neurons were obtained from the various cohorts. We utilised *n* = 309 of these for mtDel/mtCN analysis; by cohort, this amounted to *n* = 103 (16.8 ± 0.3) for the neurological‐controls; *n* = 98 (15 ± 0.2) for DLB cases, and *n* = 108 (19.2 ± 0.5) for PDD cases; values in brackets show the average (±SEM) for individual cases grouped by cohort. The remainder (*n* = 228) of the LC‐noradrenergic neuronal pool was utilised for the single‐cell nuclear‐encoded gene expression assay and was stratified per cohort as follows: *n* = 72 for neurological‐controls (6.7 ± 0.1); *n* = 83 for DLB cases (6.2 ± 0.1), and *n* = 73 for PDD cases (6 ± 0.1).

### 2.3 Multiplex mtDel and mtCN Assay

mtDel and mtCN levels per individual PPN‐cholinergic and LC‐noradrenergic neuron were quantified via a multiplex TaqMan quantitative polymerase chain reaction (qPCR) assay; Table S2 lists qPCR primer and probe sequences. The method compares intracellular levels of two mitochondrial genes, *MTN‐D1* and *MTN‐D4*, relative to a standard curve (Bury et al. 2017). *MTN‐D4* locates within the mitochondrial genome's ‘major arc’, between the heavy and light strands' origins of replication, commonly impacted by somatic mtDels (Bury et al. 2017).

### 2.4 Nuclear‐Encoded Gene Expression

Real time qPCR *TFAM*, *PGC1α* and *PINK1* primer‐probes were designed using PrimerQuest (https://eu.idtdna.com/pages/tools/primerquest) (Table S2). RNA was purified from single post‐mortem PPN‐cholinergic and LC‐noradrenergic neurons (PicoPure RNA‐isolation kit, Thermo‐Fisher). The reaction (98°C for 1 min; 30‐cycles of 98°C for 30 s; 63°C (*TFAM* and *PGC1ɑ*); 61°C (*PINK1*) for 30s and 72°C for 60 s) was performed in 96‐well plates using a Lightcycler‐480 instrument (Roche), with instrument software calculating CT values. Each plate contained triplicate sets of standards (100; 1000; 10,000; 100,000 copies/template/well), no‐template negative controls and test samples.

### 2.5 Statistics

SPSS (v.28, IBM, USA) was used for data analysis. Following analysis of variance (ANOVA), Tukey's post hoc analysis was performed. The strength of relationships between variables was determined using a Pearson's correlation test. Values are mean/median ± SEM; *p < 0.05, **p < 0.01, ***p < 0.001, ****p < 0.0001, and *p* > 0.05 (non‐significant).

## 3 Results

### 3.1 DLB and PDD‐Affected PPN‐Cholinergic Neurons Harbour High mtDel Levels With Reduced mtCN Seen in DLB

mtDNA somatic mutations manifesting as missing genomic segments (mtDels) associate with neuronal death during age‐related neurodegenerative disease (Valiente‐Pallejà et al. 2022). Errors during mtDNA replication underlie formation and clonal expansion of somatic mtDels (Elson et al. 2001). Since mtDNA mutants are recessive, mtDels must be the dominant genotype (> 60%) before cells manifest severe OXPHOS defects, potentially triggering apoptosis (Bury et al. 2017; Elson et al. 2001).

**FIGURE 1:**
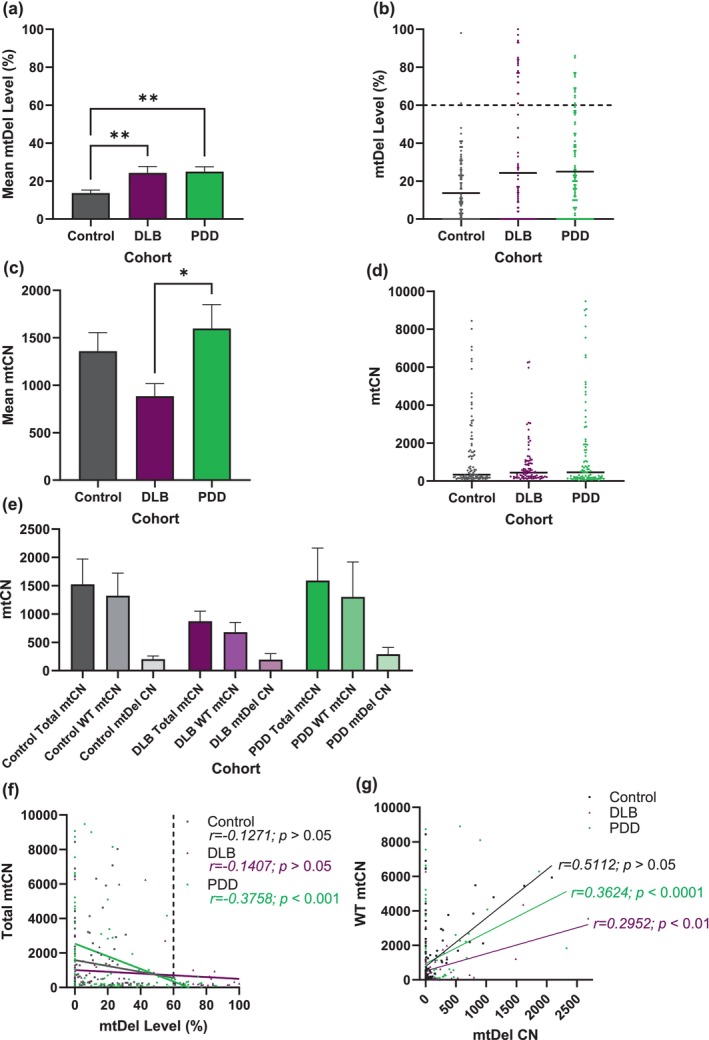
mtDel load characterises PPN‐cholinergic neurons of both LBD subtypes but mtCN change is DLB‐specific. (a) Neurotype‐specific single cell analysis revealed that DLB and PDD‐affected cholinergic PPN neurons harbour significantly greater mtDel burden compared to neurological‐control post‐mortem neurons (**p < 0.001). (b) A data scatter plot reveals how individual neurons from both LBD cohorts frequently surpassed the 60% threshold for inducing mitochondrial dysfunction, compared to control cases (***p < 0.0001). Marginally more (8%) DLB than PDD‐affected PPN‐cholinergic neurons harboured > 60% mtDel levels. (c) Total mtCN was depleted in DLB‐affected neurons compared to PDD ones (*p < 0.05), where mtCN was maintained (versus control; *p* > 0.05). (d) Dots indicate mtCN data points for individual neurons; value ranges: 67–32,341 (controls), 99–21,182 (DLB) and 30–41,902 (PDD). (e) A data figure showing the triad of measures for drawing these conclusions: Total mtCN, WT mtCN and mtDel‐harbouring mtCN. WT mtDNA was maintained in PPD but not in DLB. (f) Correlative analysis revealed a decline in total mtCN as mtDel % increased, reaching statistical significance for PDD (***p < 0.001). We previously reported a similar effect for PPN‐cholinergic neurons in post‐mortem PD biopsies (Bury et al. 2017). (g) The notion that WT mtCN is a better metric of a cell's OXPHOS capacity than mtDel levels is gaining traction. Correlative analysis which included WT mtCN as a factor revealed an attempt to maintain WT mtCN in at least some PPN‐cholinergic neurons for both DLB (**p < 0.01) and PDD cases (***p < 0.001). Few neurons linearly expressed concomitant WT mtCN and mtDel increases in DLB. Thus, the LBD subtype's disease mechanism seemingly co‐depends on the neuronal pool attempting ‘maintenance of mtDNA wild‐type’ for survival such neurons, the latter being inversely affected by DLB's relatively short DoD. Each dot represents values obtained for (b) mtDel and (d) mtCN measurements per individual neuron.

mtDel levels of single PPN‐cholinergic neurons showed that DLB (**p < 0.01) and PDD patients (**p < 0.01) contain significantly higher mtDel levels compared to controls, with similar mtDel levels recorded between disease cohorts (DLB: 24.32 ± 3.39, PDD: 24.98 ± 2.52; *p* > 0.05; Figure 1a). DLB (*n* = 21 of 94; 22%) and PDD (*n* = 14 of 101; 14%) patients had considerably more neurons containing mtDNA damage levels that exceeded 60%, a threshold level associated with a respiratory chain deficiency, compared to only 2% (*n* = 2 of 107) of control case neurons that surpassed this mtDel level (Figure 1b). However, case‐by‐case data revealed that not all DLB (3 out of 6)/PDD (3 out of 5) patients had more PPN‐cholinergic neurons that harboured high mtDel levels (Figure S3a). Mean mtCN was lower in DLB compared to PDD (*p < 0.05; Figure 1c) but differed non‐significantly between DLB versus control and PDD versus control. No significant difference existed between individual cases' mean mtCN values (Figure 1d), although more stratification was observed between diseased than neurological‐controls (Figure S3b).

It remains unclear whether defective OXPHOS results from loss of WT mtDNA, mutated mtDNA, or both (Bury et al. 2017). Our results relating to LBD‐subtype affected PPN‐cholinergic neurons implies disruption to mechanisms that allow neurons to ‘maintain wild‐type’ of mtDNA, for meeting cellular energy requirements (Elson et al. 2001). We calculated the proportion of WT mtDNA molecules as WT mtDNA copies = Total mtCN–mtDels. DLB‐ (24.32%) and PDD‐affected PPN‐cholinergic neurons (24.98%) harboured similar mtDel levels. However, WT mtDNA levels were lower (52%) in DLB‐impacted neurons versus PDD ones (*p* > 0.05; Figure 1e); the PDD cohort's mean level was comparable to neurological‐controls (*p* > 0.05). Hence, for this vulnerable neuronal population, ‘maintenance of mtDNA wild‐type’ occurred in PDD, but lacked in DLB (Figure 1e).

The relationship between accumulating %mtDels and total mtCN in LBD subtype‐specific PPN‐cholinergic neurons showed a significant negative correlation (***p < 0.001) in PDD‐affected PPN‐cholinergic neurons, indicating an absent mtCN response to accumulating mtDels; no correlation for this neuronal population was seen in control or DLB patients (Figure 1f). However, WT mtCN correlated against mtDNA copies containing mtDels showed WT mtCN upregulation to maintain mtDNA homeostasis in some PPN‐cholinergic neurons. PDD revealed pronounced upregulation, but few DLB‐affected neurons linearly increased WT mtCN relative to accumulating mtDels (DLB: **p < 0.01, PDD: ***p < 0.001; Figure 1g). Therefore, the ability to turn over WT mtCN to recalibrate against mtDel increases was maintained in this neurotype in PDD, but severely impaired in DLB cases.

### 3.2 DLB‐Affected LC‐Noradrenergic Neurons Harbour High mtDel Levels While PDD Ones Show mtCN Depletion

**FIGURE 2:**
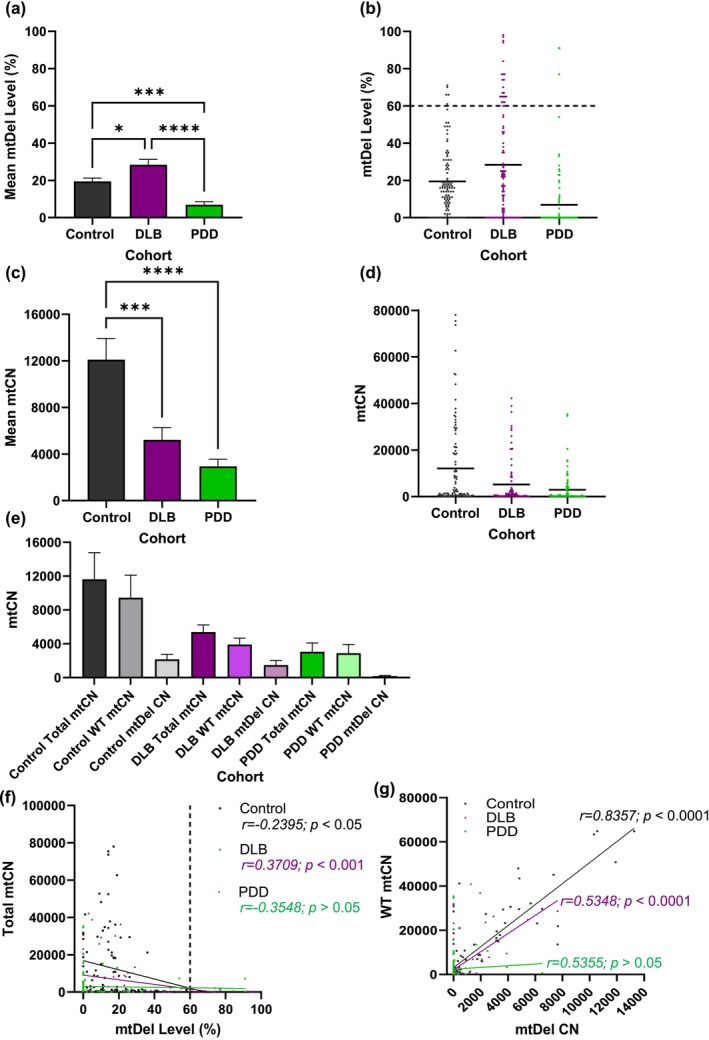
LBD subtypes non‐discriminate mtCN depletion but mtDel increased in DLB LC‐noradrenergic neurons only. (a) Compared to neurological controls, LC‐noradrenergic single‐cell analysis revealed that only DLB‐affected neurons harbour significantly greater mtDel load (*p < 0.05); PDD‐affected neurons held few mtDels (****p < 0.0001). (b) A data scatter plot corroborates this result, revealing that 22 out of 98 (22%) individual DLB neurons surpassed the OXPHOS deficiency threshold. Only 2 out of 108 (2%) PDD‐affected neurons were > 60% mtDels, less than seen in control cases (5%). (c) mtCN depletion affected both DLB (***p < 0.001) and PDD patients (****p < 0.0001) in this neurotype compared to control cases; the reduction was most pronounced in PDD (43% lower compared to DLB). (d) mtCN data points for individual neurons are indicated by dots. Similar to PPN‐cholinergic neuronal values, LC‐noradrenergic values ranged significantly: 40–99,160 for control, 47–111,960 for DLB, and 58–172,532 for PDD. (e) The data stratified as total mtCN, WT mtCN and mtCN harbouring mtDels suggests the noradrenergic neurons' inability to maintain WT mtDNA when affected by either DLB or PDD. This also reveals that the near absence of mtDel levels coupled with severe mtCN depletion are striking features of this pontine neuronal type in PDD patients. (f) A correlation analysis comparing total mtCN against mtDels (%) revealed a reduction in overall mtDNA content as mtDNA levels (%) increased for both control (*p < 0.05) and DLB (***p < 0.001). (g) When the variables of interest were WT mtCN, critical for maintaining cellular homeostasis and absolute mtDel levels, results revealed a rise in WT mtCN due to increased single‐neuronal mtDels in both control (****p < 0.0001) and DLB cases (****p < 0.0001). This relationship was absent in PDD‐affected neurons, potentially due to very low mtCN and mtDels which characterised LC NA neurons of this cohort. Each dot represents values obtained for (b) mtDel and (d) mtCN measurements per individual neuron.

Compared to DLB, PDD‐impacted LC‐noradrenergic neurons revealed low mtDel levels (****p < 0.0001), which were even lower than in controls (***p < 0.001; Figure 2a). Stratification of LC‐noradrenergic mtDel data revealed that 22% of single neurons analyzed from DLB brains contained mtDel levels at > 60% (Figure 2b). Only three single PDD‐impacted LC‐noradrenergic neurons (of 108; 3%) reached > 60% mtDel levels, compared to much larger numbers of DLB‐affected neurons (*n* = 22 of 98; 22%) and even control cases' LC‐noradrenergic neurons (*n* = 5 of 103; 5%) (Figure 2b). Inspection of mtDel LC‐noradrenergic data in individual cases corroborated this result, with some neurons harbouring > 60% mtDel levels in most DLB cases, whereas only three PDD cases harboured mtDel levels that superseded this threshold (Figure S3c).

Compared to neurological controls, profound mtDNA depletion characterised LC‐noradrenergic neurons of DLB cases (***p < 0.001), which was even more prevalent in PDD (****p < 0.0001; Figure 2c,d); case‐by‐case mtCN data revealed cohort‐dependent consistency (Figure S3d). When mtCN data was stratified to determine whether LC‐noradrenergic neurons harboured similar levels of mtDNA copies containing large‐scale mtDels (DLB: 24.32%, PDD: 24.98%), visual representations indicate that DLB and PDD‐affected LC‐noradrenergic neurons contain lower levels of WT mtDNA molecules compared to controls (Figure 2e).

Total mtCN against mtDels revealed a significant decline in mtDNA content as mtDel levels increased, for control (*p < 0.05) and DLB cases (***p < 0.001; Figure 2f). However, WT mtCN (as opposed to total mtCN) correlated against absolute mtDel levels (as opposed to %mtDels), resulting in a statistically significant positive correlation between these parameters in both control (****p < 0.0001) and DLB cases (****p < 0.0001; Figure 2g). Since mtCN in this neurotype for DLB cases was substantially less than control cases, this may reflect an attempt by a proportion of LC‐noradrenergic neurons to maintain WT, whilst the ability was lost by such remaining neurons in DLB. In PDD‐affected LC‐noradrenergic neurons, no such relationship between mtDel‐mtCN and WT‐mtCN was seen (*p* = 0.58; Figure 2g).

mtDel levels were marginally higher in DLB‐affected LC‐noradrenergic neurons compared to mtDel load of the same cohort's PPN‐cholinergic neurons (*p >* 0.05) but was significantly higher in LC‐noradrenergic than PPN‐cholinergic neurons of neurological‐control cases (*p < 0.05). Interestingly, non‐diseased LC‐noradrenergic neurons harboured ~10‐fold higher mtCN per neuron (12,107 ± 1814; Figure 2c) than was measured in PPN‐cholinergic neurons (1359 ± 196, ****p < 0.0001; Figure 1c), suggesting that LC‐noradrenergic neurons may have higher inherent ATP requirements than that of PPN‐cholinergic neurons. From this baseline, a far greater reduction in mtCN was observed in DLB‐affected LC‐noradrenergic (5211 ± 1055; Figure 2c,d) than PPN‐cholinergic neurons (884 ± 134; Figure 1c,d). Compared to control brains, a highly pronounced mtCN reduction characterised PDD‐affected LC‐noradrenergic neurons (****p < 0.0001; Figure 2c,d). This feature appeared pontine neurotype‐specific, since such cases' PPN‐cholinergic neurons lacked a distinct mtCN change, with mean mtCN levels being comparable to neurological‐controls' LC‐noradrenergic neurons (*p* > 0.05; Figure 1c,d). These observations indicate that the rules governing mtDNA maintenance differ between these pontine neurotypes, the underlying neural pathology inherent to the disease subtype influencing how clonal expansion of mtDels manifests.

### 3.3 Correlative Relationships Between mtDNA Metrics and LBD'S Duration of Disease

**FIGURE 3:**
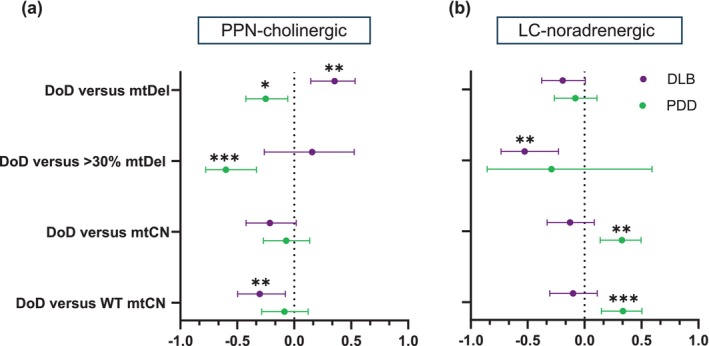
Correlative mtDNA‐DoD for both pontine neurotypes‐of‐interest in LBD subtypes. (a) When considering PPN‐cholinergic neurons, mtDels showed a significant positive relationship with DoD in DLB (*r* = 0.3546, **p < 0.01), but the correlation was negative in PDD (*−r* = 0.2502, *p < 0.05). A strong negative relationship was seen for neurons with > 30% mtDels and DoD in PDD (*r* = −0.599, ***p < 0.001). WT mtCN correlated negatively with DoD in DLB (*r* = −0.3026, **p < 0.01). (b) Within single LC‐noradrenergic neurons, no significant correlation was seen in either disease cohort between mtDels (%) and DoD, but a significant negative relationship was detected in DLB between mtDel levels with pathogenic potential (> 30%) and DoD (*r* = −0.5262, **p < 0.01). In PDD, mtCN correlated highly positively with DoD (*r* = 0.3279, **p < 0.01), revealing an even stronger positive correlation with WT mtCN (*r* = 0.339, ***p < 0.001).

Figure 3 summarises the results of Pearson's correlation analyses between mtDNA measures and duration of disease (DoD). DoD data was available for 9 (out of 10) DLB cases and all PDD cases (Table S1). Correlating with published observations (Larsson et al. 2018), the PDD patients' mean DoD was substantially longer (22 ± 3.6 years) compared to DLB cases (6.4 ± 1.9 years; *p < 0.05). DLB patients' DoD correlated significantly with mtDel load harboured by PPN‐cholinergic neurons (*r* = 0.4, **p < 0.01). The same analysis produced a significant negative relationship for PDD patients (*r* = −0.3, *p < 0.05), implying that the long DoD allows for adaptive compensations by mtDNA to sustain the mtDel levels. The correlation analysis was repeated after excluding neurons containing mtDels < 30%, since neuroprotective processes are unlikely to be triggered at lower mtDel levels (Pyle et al. 2016). The results revealed a positive, but non‐significant, relationship between PPN‐cholinergic neurons containing higher mtDel levels and DoD in DLB cases (*p* > 0.05) whilst an extremely significant negative correlation was seen for PDD cases (*r* = −0.6, ***p < 0.001), corroborating our hypothesis that DLB's extended DoD instigates compensation against mtDels' clonal expansion.

Non‐significant DoD‐mtCN relationships were discerned for PPN‐cholinergic neurons of DLB and PDD cases. However, a significant negative relationship was detected between WT mtCN and DLB patients' DoD (*r* = −0.3, **p < 0.01). A negative, albeit non‐significant relationship (*p* > 0.05) existed for PDD, suggesting that in PPN‐cholinergic neurons, mtDNA WT copies are better maintained over the disease course in PDD than DLB patients.

In LC‐noradrenergic neurons, no associations were seen when the effector was overall mtDel level (DLB: *r* = −0.2, *p* > 0.05; PDD: *r* = −0.1, *p* > 0.05), whilst revealing significance for DLB only when mtDel levels with pathogenic potential (> 30%) were considered (*r* = −0.5, **p < 0.01, PDD: *r* = −0.3, *p* > 0.05). Correlative analysis revealed opposing trends between DLB and PDD cases for mtCN‐DoD relations, showing non‐significance for DLB, but a highly significant positive relation in PDD (*r* = 0.3, **p < 0.01), suggesting that expanding levels of mtDels per LC‐noradrenergic neuron over PDD's protracted DoD may be a key driver for such neuron's apoptosis. No clear relationship was seen between the proportion WT mtDNA copies and DoD in DLB (*r* = −0.1, *p* > 0.05). However, for PDD cases, a strong positive relation existed (*r* = 0.3, ***p < 0.001), implicating WT mtDNA biogenesis as a countermeasure against rising mtDel levels, potentially playing a role in the relatively longer survivability of PDD compared to DLB patients.

### 3.4 mtDNA Variance Triggers Pontine Neurotype‐Dependent Nuclear‐Regulating Pathway Responses During PDD

**FIGURE 4:**
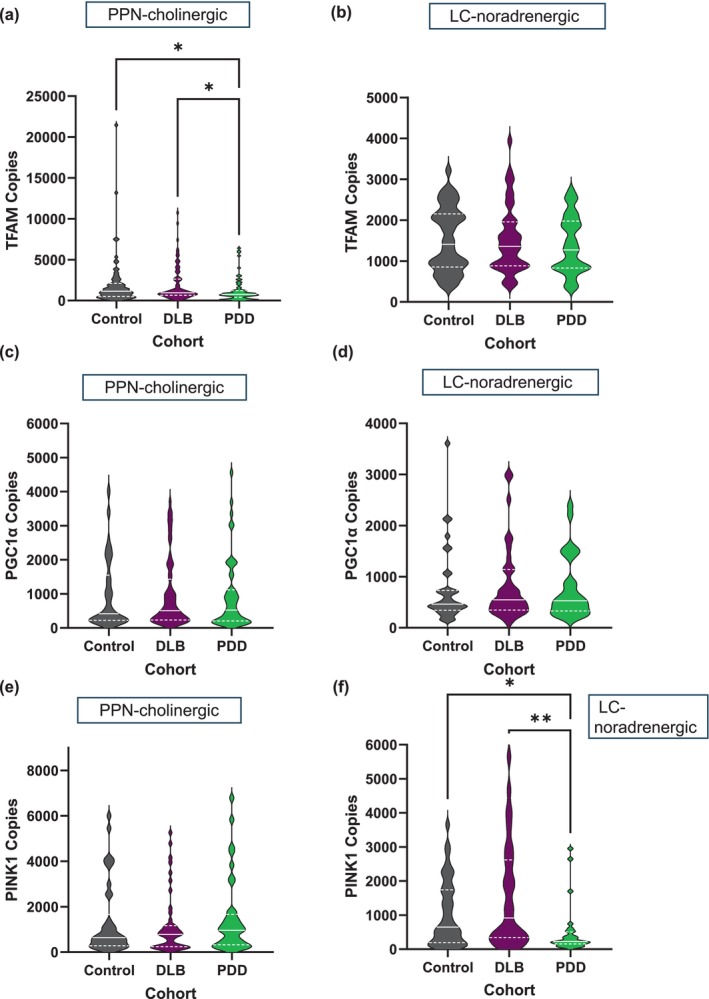
Violin plots to reveal comparative median expression levels of nuclear‐encoded genes intended for maintaining mtDNA stability. (a) Comparative analysis revealed significantly decreased *TFAM* expression in PPN‐cholinergic neurons of the PDD cohort (median: 762; lower quartile (LQ): 394; upper quartile (UQ): 1215) compared to both DLB (median: 947; LQ: 654; UQ: 2620; *p < 0.05) and control cases (median: 1138; LQ: 506; UQ: 2099; *p < 0.05). (b) In comparison, no significant median *TFAM* mRNA level changes were discerned between the cohorts in LC‐noradrenergic neurons. (c) Median *PGC1α* mRNA levels were highly similar (*p* > 0.05) between the cohorts when measured in PPN‐cholinergic, (d) and LC‐noradrenergic neurons. (e) This contrasted with the absence of notable PPN‐cholinergic *PINK1* expression level differences between the cohorts (*p* > 0.05), (f) such mRNA expression was severely depleted within LC‐noradrenergic neurons of PDD cases (median: 227; LQ: 155; UQ: 507; **p < 0.01) compared to both DLB (median: 227; LQ: 155; UQ: 507) and control cases (median: 651; LQ: 192; UQ: 1743).

We studied whether the observed changes in mtDNA quantity and quality had introduced mtDNA‐regulating nuclear gene recalibrations. In PPN‐cholinergic neurons, PDD cases showed reduced mRNA levels of the mtDNA maintenance gene, *TFAM*, compared to DLB (61.8%, *p < 0.05) and neurological‐control cases (60.2%, *p < 0.05; Figure 4a). *TFAM* mRNA reduction was specific to PPN‐cholinergic neurons for PDD patients, with no expression level change noted between the cohorts for LC‐noradrenergic neurons (Figure 4b). It is noteworthy that significantly lower levels of *TFAM* mRNA copies were seen in single LC‐noradrenergic compared to individual PPN‐cholinergic neurons, a consistent observation per neurotype, regardless of whether measures occurred in neurological‐control (PPN‐cholinergic:1961 ± 37.7; LC‐noradrenergic: 1520 ± 16.4), DLB (PPN‐cholinergic: 1910 ± 28.1; LC‐noradrenergic: 1526 ± 17.7) or PDD‐derived neurons (PPN‐cholinergic: 1181 ± 19.2; LC‐noradrenergic: 1392 ± 18).

We anticipated *TFAM* mRNA reduction as an adaptive response to counteract altered expression of the mitochondrial biogenesis stimulator, *PGC1α*, since *TFAM* is a downstream effector of *PGC1α*'s signalling cascade (Nissanka and Moraes 2018). However, *PGC1α* mRNA levels were unaltered between cohorts for both neurotypes (PPN‐cholinergic, Control: 981 ± 17.5; DLB: 973 ± 15.8; PDD: 943 ± 20.4; *p* > 0.05; Figure 4c; LC‐noradrenergic, Control: 742 ± 19.3; DLB: 825 ± 19.2; PDD: 769 ± 17.9; *p* > 0.05; Figure 4d).

Similar expression levels of *PINK1* manifested in PPN‐cholinergic neurons between cohorts (Control: 1351 ± 42; DLB: 1148 ± 28.5; PDD: 1492 ± 45; *p* > 0.05; Figure 4e) but were notably reduced in PDD's LC‐noradrenergic neurons (502 ± 27.4) compared to controls (1057 ± 23; *p < 0.05) and DLB (1552 ± 48.6; **p < 0.01; Figure 4f). Figure S4 provides case‐by‐case mRNA data scatter plots.

### 3.5 Nuclear Gene Transcriptome Changes Predict Disease Duration in DLB PPN Cholinergic Neurons

**FIGURE 5:**
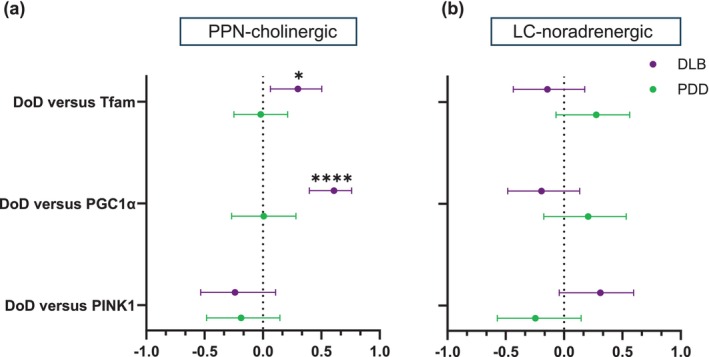
mtDNA‐regulating genes' mRNA levels and disease course metric correlations in pontine cholinergic and noradrenergic neurons. Bivariate Pearson's correlation analysis was applied to single (a) PPN‐cholinergic and (b) LC‐noradrenergic neurons to interrogate the relationship between AaD and DoD and the mRNA levels of three genes. The dot intersecting each summary line indicates the *r*‐value, whilst the parallel bars visually represent the level of uncertainty relating to this correlation coefficient value. Positive correlation effects transcend towards the right of the perforated central line, whilst a negative correlation transcends towards the left. (a) *TFAM* expression levels exhibited a marginally significant positive relationship with AaD (*r* = 0.2828, *p < 0.05) in the DLB cohort. *PGC1α* demonstrated a significant negative correlation with this variable in the control (*r* = −0.3905, **p < 0.05) and PDD groups; there was a marginal positive relationship (*r* = 0.272, *p < 0.05). Only DLB cases showed a significant positive association between *PINK1* levels and AaD (*r* = 0.312, *p < 0.05). No significant connections were seen between the gene expression data and DoD for PDD in this neurotype. However, DLB cases showed a *TFAM*‐DoD positive correlation (*r* = 0.299, *p < 0.05) whilst a *PGC1α*‐DoD correlation (*r* = 0.6085, ****p < 0.0001) was especially significant. (b) Within single LC‐noradrenergic neurons, the *TFAM* expression levels exhibited a marginally significant positive relationship with AaD in the control cohort (*r* = 0.3215, *p < 0.05), with a stronger negative relationship in DLB cases (*r* = −0.4, **p < 0.01). No significant associations were detected between the targeted genes and DoD in either disease cohort.

To characterise relationships between the nuclear‐encoded genes of interest and DoD, we conducted bivariate Pearson's correlation analyses (Figure 5). Positively directed associations were seen in DLB cases' PPN‐cholinergic neurons relating to *TFAM* (*r* = 0.3, *p < 0.05) and *PGC1α* CN (*r* = 0.6, ****p < 0.0001). Neither *TFAM* nor *PGC1α* levels were significantly altered in this neurotype during DLB (Figure 4a,c). This suggests that these genes' transcription is subject to a dynamic time order, with more resilient, stably surviving PPN‐cholinergic neurons possibly harbouring more enriched mRNA at advanced disease stages, either due to the positive selection of a less vulnerable long‐term surviving PPN‐cholinergic neuronal subtype or up‐regulation of neuroprotective gene products. No notable associations were observed between gene transcript levels and DoD in LC‐noradrenergic neurons.

## 4 Discussion

LBD‐affected brains show progressive neuronal degeneration affecting specific neuronal populations, correlating with disease severity. A more profound loss of cholinergic neurons occurs in PDD and DLB compared to PD and AD (Francis and Perry 2007). Relatedly, cholinesterase‐inhibiting drugs offer symptomatic treatment (Li et al. 2019). During LBD, the noradrenergic LC also degenerates, exceeding levels in PD brains (German et al. 1992).

We interrogated two pontine‐based neurochemical neurotypes in clinically differentiated DLB versus PDD post‐mortem brains. We evidence that neurotype‐specific mtDNA defects are a primary molecular‐pathological mechanism underlying LBD syndromes' differential phenotypes. Our findings diverge from the consensus that end‐stage DLB and PDD are similar (Donaghy and McKeith 2014), instead revealing mtDNA‐related disease mechanistic differences that manifest neurotype‐specifically.

We unravelled a key neuropathological hallmark relating to mtCN changes, with different patterns seen between LBD subtypes and neurotypes. Moreover, differential abilities to maintain WT mtDNA were discerned, with DLB patients' PPN‐cholinergic neurons showing greater deficiency. Finally, we addressed whether prominent genes regulating mtDNA maintenance (*TFAM*), biogenesis (*PGC1α*) and mitophagy (*PINK1*) underlie the observed mtDNA variance characterising pontine neurotypes and LBD subtypes. We revealed altered expression of *TFAM* and *PINK1* in both neurotypes of PDD but not DLB cases, suggesting that disrupted nuclear‐mitochondrial synergism drives the diverse mtDNA pathological patterns seen between LBD syndromes.

**FIGURE 6:**
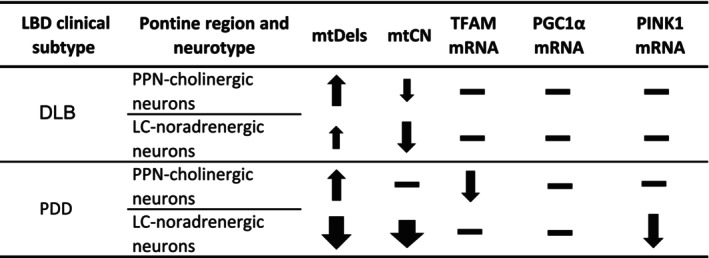
Summary of the current mtDNA variance and key nuclear‐encoded mtDNA regulating genes expression profile results. Arrows indicate the direction of change, the size depicting the degree of change observed in the patient cohorts, relative to neurological control patients.

Our results, summarised in Figure 6, reveal prominent differences between LBD phenotypes relating to mtDel accumulation within pontine neurons, adding credence to the notion that mtDels cause neuronal harm, manifesting as LBD syndrome‐differentiating disease symptoms. High mtDel levels affected PPN‐cholinergic neurons of both LBD syndromes compared to neurological controls. For DLB cases, mtDel levels were similar between the two neurotypes of interest but, intriguingly, very low mtDel levels were detected in PDD cases' LC‐noradrenergic neurons, even below that of neurological‐controls. The post‐mortem brain tissue utilised here had been retrieved from DLB and PDD cases that were disease end‐stage, consensus dictating that disease pathology largely overlaps at the terminal time‐point (Donaghy and McKeith 2014). However, considering the profoundly different clinical phenotypes that DLB versus PDD patients present with during early disease stages, it is feasible that LC‐noradrenergic neurons might have incurred significant mtDNA damage during initial disease, when symptom profiles between DLB and PDD are more distinct. This may have instigated substantial LC‐noradrenergic cell death, leaving only a fraction remaining at disease end‐stage, appearing unaffected by mtDels' clonal expansion. For this pontine neuronal population, this implies that LBD syndromes differ i.t.o. the critical time point for vulnerable neurons to sustain mtDel damage, with DLB‐affected neurons possibly suffering a more protracted neuronal insult.

mtDNA continuously replicates independent of the cell cycle (St John 2016), making mtCN an indicator of a cell's competence to utilise OXPHOS for meeting neurons' high energy requirements. We observed similar mtCN responses to the high mtDel load in both DLB‐affected neurotypes. Strikingly, the mtCN profile in PDD‐affected LC‐noradrenergic neurons resembled autosomal‐recessive mtDNA depletion syndrome (MDS)/Alper's disease, characterised by cellular mtDNA depletion (Rusecka et al. 2018).

We previously conducted single‐cell analyses of post‐mortem PD‐affected PPN‐cholinergic neurons (Bury et al. 2017), revealing mtDNA differences between PD‐susceptible mesencephalic‐dopaminergic neurons compared to PPN‐cholinergic ones. Both neurotypes harboured high levels of large‐scale mtDels in PD compared to controls. Additionally, in response to PD‐induced mtDel accumulation, mtCN changes manifested neurotype‐specifically: Vulnerable dopaminergic neurons showed mtCN depletion (Dölle et al. 2016), whilst PD‐affected PPN‐cholinergic neurons showed elevated mtCN compared to controls (Bury et al. 2017). The elevation may represent a cellular ability to proliferate mtDNA to maintain a critical number of WT mtDNA molecules to support neuronal energy requirements. Whether mtDels' pathogenic effects result from mutations or lack of WT mtDNA evokes intense debate (Elson et al. 2001). Although WT mtDNA was maintained or at least did not significantly decline during PDD, this compensation was near‐absent in DLB, potentially reflecting differences in LBD subtypes' time course. DLB patients typically exhibit a relatively short period to reach patho‐phenotypic endpoints, post‐diagnosis survival time being ~3 years, whilst a more protracted disease trajectory is usual for PDD patients, post‐diagnosis median survival time being 9 years (Larsson et al. 2018). Our results showed a strong inverse linear relationship between WT mtCN and DoD for DLB cases' PPN‐cholinergic neurons, implying that a longer DoD results in reduced levels of non‐mutated mtDNA molecules. A similar decline in WT mtCN was lacking in PDD‐affected PPN‐cholinergic neurons, suggesting that WT is maintained, or at least that there is not a significant decline in WT mtCN of such neurons. Considering DLB's typically short DoD, this suggests greater clonal expansion of mtDels for driving DLB patients' more severe phenotype compared to PDD. Hence, the less aggressive disease course of PDD, where disease‐susceptible neurons potentially survive longer than during DLB, likely relates to PDD‐affected neurons' ability to maintain WT more readily than occurs in DLB.

For progressing towards therapeutic options, future work should discern the role played by failure of this homeostatic feedback mechanism for maintaining WT mtDNA levels, and how this may cause PPN‐cholinergic neuronal loss in DLB brains. No correlative relations were discerned between DoD and any mtDNA metrics in LC‐noradrenergic neurons, suggesting that DLB's more acute DoD prevents pathological mtDel levels from accumulating within such neurons. Hence, PDD's protracted DoD predicts that LC‐noradrenergic neurons' mtDNA mutation rate, closely linked with mtDNA turnover in post‐mitotic neuronal cells (St John 2016), occurs slower than in such neurons' of DLB brains.

nDNA encodes all proteins regulating mtDNA maintenance, ‐replication, ‐transcription and mitophagy (Ali et al. 2019). To investigate which mtDNA‐regulating gene pathway may be recruited to counteract the observed LBD subtype‐dependent mtDNA damage, we undertook neurotype‐specific single‐cell multi‐gene‐copy analysis. We identified a prominent *TFAM* mRNA decrease in PPN‐cholinergic neurons from PDD patients compared to DLB and controls. *TFAM* is a core component of the mitochondrial transcription initiation machinery, whilst also packaging mtDNA into mitochondrial nucleoids (Larsson et al. 1998). Insights as to bidirectional *TFAM*‐mtDNA regulation derive mainly from manipulating *TFAM* expression to determine effects on mtDNA, thereby assuming that this cause‐effect relationship is driven linearly by *TFAM*. For instance, early work by Larsson et al. (1998) utilised mice lacking *TFAM* to demonstrate that impaired mtDNA transcription and the inability to maintain mtDNA result in bioenergetic failure and mtDNA depletion affecting the heart tissue. More recent work reduced *TFAM* copies in zebrafish embryos via a splice‐modifying morpholino, which decreased mtCN concomitant with abnormal development of the eye, brain, heart, and skeletal muscles, as well as decreased ATP production (Otten et al. 2020). In other work, Nguyen et al. (2024) generated *TFAM* heterozygous knockouts via CRISPR (Clustered Regularly Interspaced Short Palindromic Repeats)‐Cas9 in human embryonic kidney cell lines, resulting in reduced mtCN. However, our PPN‐cholinergic neuronal analyses question such TFAM‐driven retrograde communication with mitochondria to suggest that such signalling direction may depend on the cell type and the disease context. In this regard, in our study mtCN was intact in PDD‐affected PPN‐cholinergic neurons, yet these neurons displayed severely downregulated expression of *TFAM*. This aligns with previous reports where *TFAM* polymorphisms are associated with susceptibility to PDD but not DLB, alongside severe mtCN reduction in post‐mortem prefrontal cortices of PDD patients, suggesting that *TFAM* alteration effects are brain wide (Gatt et al. 2013). However, our results suggest that such pathological manifestations in PDD‐affected brains are governed by intrinsic neuronal features, with only certain neurotypes being affected.

*TFAM* downregulation was shown to induce reduced glycolysis rates and ATP production, threatening cell survival, whereas +/−*TFAM* heterozygous mice revealed altered mtDNA packaging that triggered the release of proinflammatory fragmented mtDNA into the cytosol, altering disease trajectory (Xie et al. 2016). Hence, *TFAM* downregulation may be a PDD‐specific disease mechanism, independent of mtDNA variance, to govern LC‐noradrenergic neurons' fate in PDD brains. This highlights the potential of therapeutic *TFAM* upregulation, shown to induce neuronal prosurvival effects (Morimoto et al. 2012).

Since our results showed LBD syndrome‐dependent *TFAM* expression changes, with *TFAM* being a downstream effector of *PGC1α*'s signalling cascade, it was surprising to find *PGC1α*'s CN unaltered, independent of LBD syndrome or neurotype. This indicates that expression changes affecting *PGC1α* in these neuronal populations of LBD subtypes are not directly causative of the observed mtCN or mtDel variation and do not responsively change due to these. Hence, the result hints at the recruitment of other nuclear‐regulated genetic cascades. Future work should aim to identify candidates using single‐cell global transcriptomic strategies, whereas post‐translational events that may have affected PGC1α protein in these pontine neurotypes, and distinguishing between LBD phenotypes, should also be investigated.

mtCN and heteroplasmy can be regulated by removing defective mtDNA via mitophagy, during which PINK1 and Parkin proteins build ubiquitin chains on damaged mitochondria' outer surface, marking them for degradation. Both mitochondrial dysfunction and nuclear‐related genotoxic stress can initiate PINK1/Parkin‐dependent mitophagy (Vives‐Bauza et al. 2010). Additionally, Gegg et al. (2009) revealed that silencing *PINK1* expression in dopaminergic SH‐SY5Y cells resulted in reduced mtCN, whilst also affecting the activity of complex IV of the mitochondrial electron transport chain (ETC) and resulting in increased markers of oxidative stress. In other work, dermal fibroblasts from a patient carrying the homozygous mutation p.W437X in *PINK1*, which results in partial loss‐of‐function of the gene to manifest as early‐onset parkinsonism, did not reveal mtCN variation (Piccoli et al. 2008). Such conflicting findings relating to correlations between *PINK1* expression changes and mtDNA content, derived from using diverse cellular models, may suggest that a direct modulatory effect by *PINK1* on mtDNA biogenesis via a mtDNA quality control mechanism is dependent on the cell type in which it occurs. In this regard, a role in disease progression was discerned where *PINK1* loss‐of‐function induced in catecholaminergic neurons showed accelerated neurodegeneration and altered catecholaminergic neurotransmitter release (Moisoi et al. 2014), suggesting that defective mitochondrial quality control might especially affect catecholaminergic (e.g., noradrenergic) neurons, thereby facilitating their neurodegeneration. We recorded distinct downregulation of *PINK1* mRNA expression, indicative of non‐functional gene signalling in PDD, but only in patients' LC‐noradrenergic neurons that also revealed significantly decreased mtCN, aligning with studies that assigned an mtDNA biosynthesis role to *PINK1* (Vives‐Bauza et al. 2010). Moreover, with mtDNA variance in LC‐noradrenergic neurons resembling MDS, MDS‐implicated nuclear genes essential for mtDNA replication for example, *Twinkle*, may occur during PDD. Studies examining how pathological signalling between *PINK1* and MDS‐associated gene pathways could drive insufficient mtDNA synthesis in such neurons during PDD may deliver therapeutic targets to counter mtDNA content reduction.

Analysis of post‐mortem brain tissue specimens from clinically well‐described patients offers unprecedented insight into the cellular and molecular changes resulting from the disease per se, contrasting with the experimental use of animal or cellular models that may not reflect authentic disease processes. However, post‐mortem tissue is limited in that it only provides a static snapshot of dynamic disease processes. Hence, follow‐up studies using reliable human in vitro cellular systems are required to establish mechanistic validation and disease causality for the results reported here, including the significantly altered mRNA level changes that manifested neurotype and LBD subtype dependently. However, to ensure that neuropathologically meaningful conclusions are derived from such investigations, studies should aim to incorporate various tiers into such experimental model tools. This entails ensuring that cell cultures are differentiated to portray a robust cholinergic versus noradrenergic pheno‐ and chemotype, and ideally also mimic the spatial architecture of the target brain regions by using co‐culture methods to include other relevant cell types (e.g., microglia and astrocytes) while being presented in a three‐dimensional format. Moreover, to mechanistically dissect the LBD‐subtype specific mtDNA and mRNA expression patterns seen here, such cell systems should ideally derive from clinically well‐characterised DLB versus PDD cases, to compare to cases free from neurodegenerative disease that is, by utilising patient‐derived dermal fibroblasts. The establishment of such cell models reflecting these key features of the brain region‐, neurotype‐ and LBD subtypes studied here combined with gene editing technologies will help decipher whether mRNA expression changes seen here are either a principal driver or a compensatory mechanism for the associated mtDNA variation reported here. Such tools can further help towards determining whether expressing gene transcripts in the reverse direction to that seen here may halt or even reverse the observed mtDNA pathological changes. Furthermore, future use of ‘multi‐omics’ transcriptome‐ and proteome‐wide technologies, applied to the DLB versus PDD target neurons, will further identify additional gene regulatory pathways recruited during the clonal expansion of mtDels and related mtCN changes. Additionally, clinical subsampling for stage‐wise representative Braak tau and Thal (amyloid plaque) phases will give insight into the extent to which high abnormal proteinaceous burden indicating AD co‐pathology may influence our reported mtDNA findings, or whether the profile discerned here is LBD‐specific.

Here we characterised mitochondrial quality control failures of two pathologically affected interconnected brainstem areas, revealing notable overlaps and distinctions between LBD subtypes, querying the medical consensus that PDD and DLB largely pathologically overlap at disease end‐stage. Using a strategy that carefully stratified LBD clinical subtypes and brainstem neurotypes, our study provides an important data foundation for future work utilising neuropathologically relevant human cell models to facilitate mechanistic interpretation of the phenomena discerned here and establish the therapeutic relevance of targeting the identified pathology‐associated mRNA expression changes. Relatedly, future work utilising prospectively acquired sample sets from cases from whom substantial clinical profiles have been collected longitudinally promises to reveal significant insight into whether mtDNA instability accelerates with progression of disease or rather represents an early disease driver.

## Author Contributions

E.J.S. and L.J.B. performed experiments. E.J.S., L.J.B., J.L.E. and I.S.P. performed data analysis. L.J.B., J.L.E., S.G. and I.S.P. wrote the first draft of the manuscript, to which all the authors provided edited contributions. All the authors read and approved the final manuscript.

## Conflicts of Interest

The authors declare no conflicts of interest.

## Supporting information


**Figure S1.** Histological stains to anatomically outline the two regions of interest.

**Figure S2.** A workflow diagram depicting key experimental steps for generating the data reported on in the current work.

**Figure S3.** (a) A data scatter plot reveals that both DLB and PDD post‐mortem brain samples contained single PPN‐cholinergic neurons which harboured significantly high mtDel levels compared to controls, with DLB‐affected samples that had a proportionally higher number of neurons that exceeded the mutation heteroplasmy threshold of 60% (by ~15%).

**Figure S4.** Data scatter plots showing *TFAM*, *PGC1α* and *PINK1* mRNA expression values of type‐specific pontine‐based neurons.

**Table S1.** Summary of the demographic, clinical and neuropathological features of the current patient and neurological‐control cases.

**Table S2.** qPCR primers and probes used in the current study.

## Data Availability

The data that support the findings of this study can be obtained from the corresponding author upon reasonable request.
