# Supplementary figures and images for: Type‐Specific Single‐Neuron Analysis Reveals Mitochondrial DNA Maintenance Failure Affecting Atrophying Pontine Neurons Differentially in Lewy Body Dementia Syndromes
Source: Aging Cell. 2025 Jun 6;24(8):e70125. doi: 10.1111/acel.70125 (PMC12341794; doi:10.1111/acel.70125)

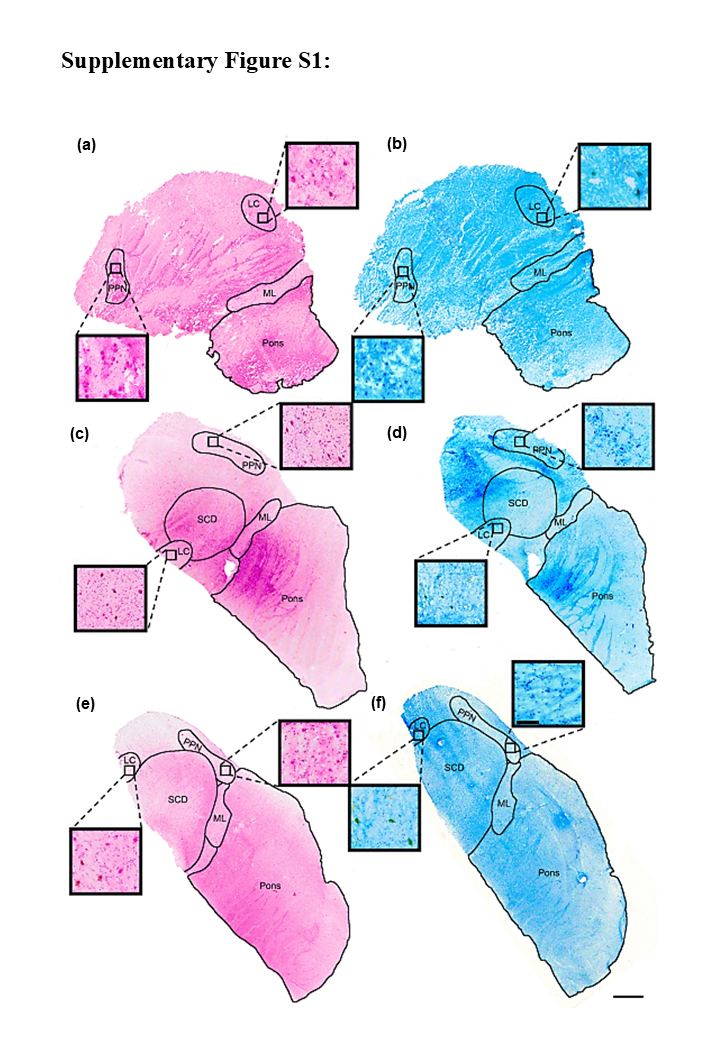

Supplement: Supplementary file 1 — Figure S1. Histological stains to anatomically outline the two regions of interest. [file ACEL-24-e70125-s005.tif]

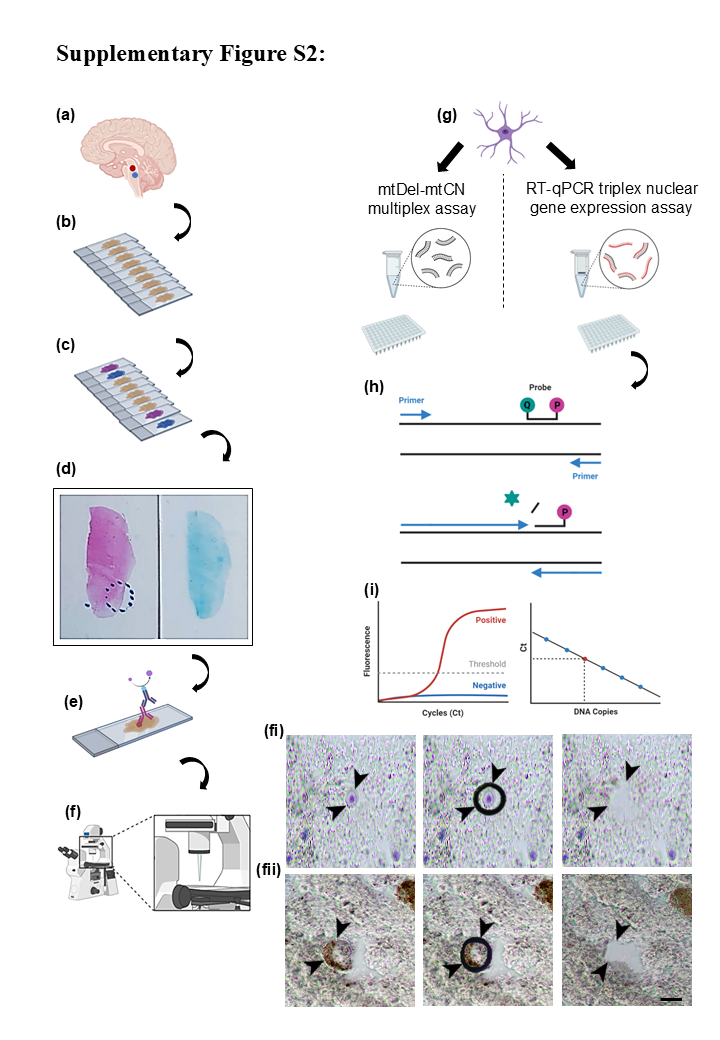

Supplement: Supplementary file 2 — Figure S2. A workflow diagram depicting key experimental steps for generating the data reported on in the current work. [file ACEL-24-e70125-s006.tif]

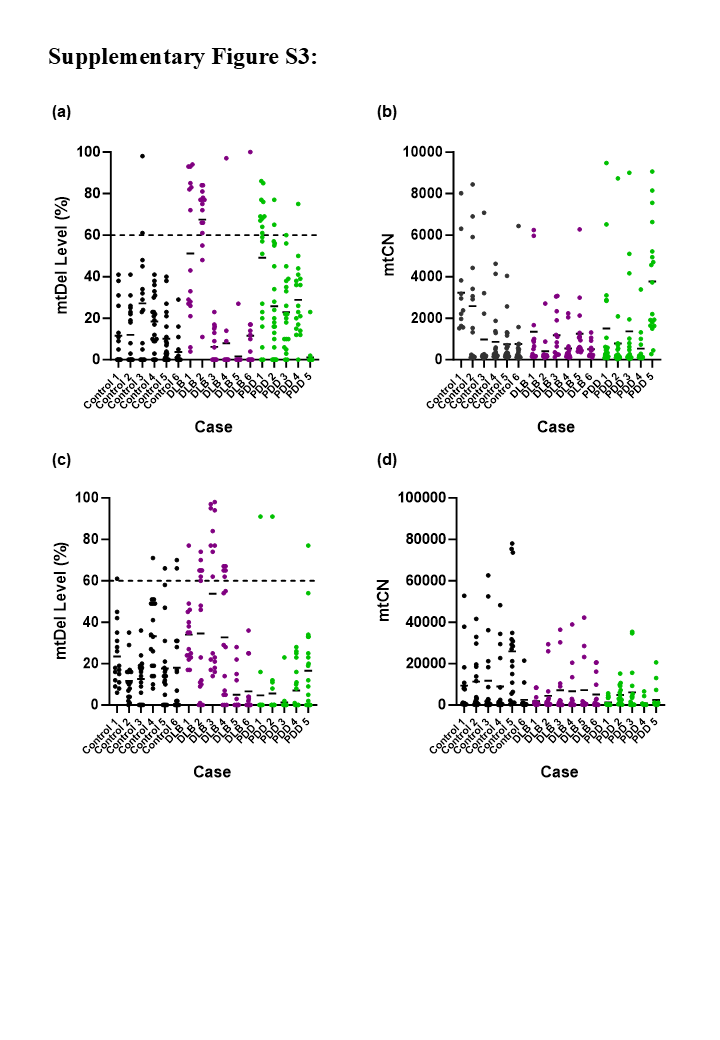

Supplement: Supplementary file 3 — Figure S3. (a) A data scatter plot reveals that both DLB and PDD post‐mortem brain samples contained single PPN‐cholinergic neurons which harboured significantly high mtDel levels compared to controls, with DLB‐affected samples that had a proportionally higher number of neurons that exceeded the mutation heteroplasmy threshold of 60% (by ~15%). [file ACEL-24-e70125-s003.tif]

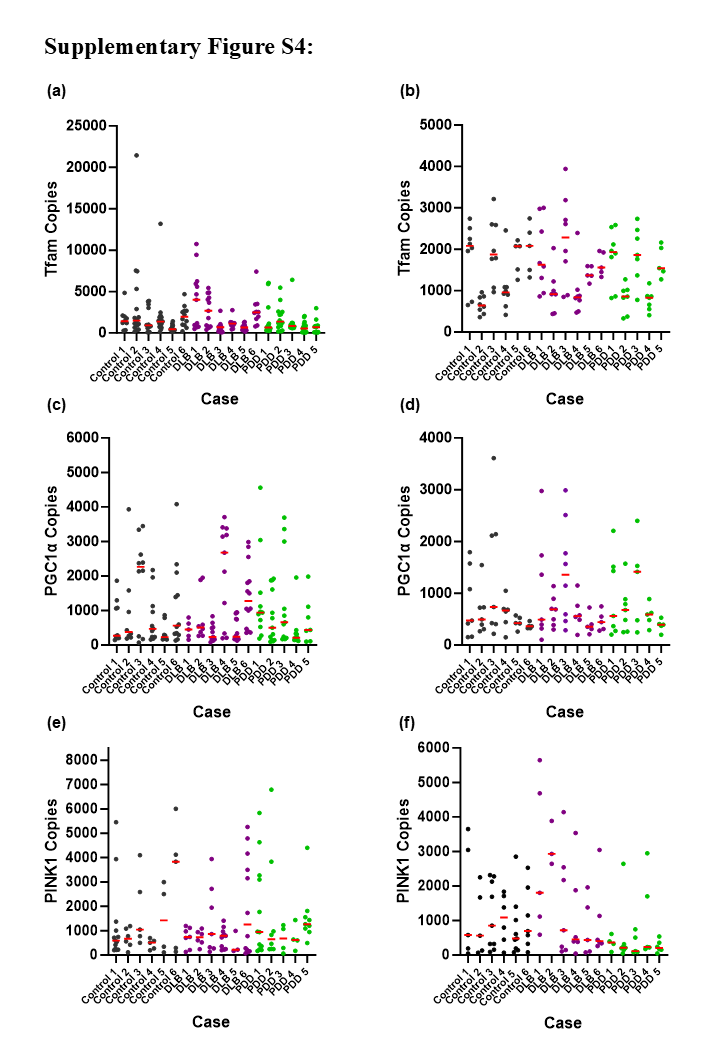

Supplement: Supplementary file 4 — Figure S4. Data scatter plots showing TFAM, PGC1α and PINK1 mRNA expression values of type‐specific pontine‐based neurons. [file ACEL-24-e70125-s001.tif]
