# Supplementary material for: Type‐Specific Single‐Neuron Analysis Reveals Mitochondrial DNA Maintenance Failure Affecting Atrophying Pontine Neurons Differentially in Lewy Body Dementia Syndromes
Source: Aging Cell. 2025 Jun 6;24(8):e70125. doi: 10.1111/acel.70125 (PMC12341794; doi:10.1111/acel.70125)
Supplement: Supplementary file 5 — Table S1. Summary of the demographic, clinical and neuropathological features of the current patient and neurological‐control cases. [file ACEL-24-e70125-s002.docx]

**Supplementary Table S1. A summary of the demographic, clinical and neuropathological features of the patient and neurological control cases included in the current study.**

| **Pontine nucleus analysed: PPN** | | | | | | | | | | | |  |
| --- | --- | --- | --- | --- | --- | --- | --- | --- | --- | --- | --- | --- |
| **Case ID (unique Brain Bank ID)** | **Sex** | **AaD (yrs)** | **PMI** | **DoD (yrs)** | **Neurological dx** | **McKeith LB stage** | **Braak LB stage** | **Braak tau stage** | **Thal phase** | **MMSE score** | **MoCA score** |  |
|  |  |  |  |  |  |  |  |  |  |  |  |  |
| CTRL 1 (BBN002.3376) | M | 73 | 19 | n/a | n/a | n/a | 0 | II | 2 | n/a | n/a |  |
| CTRL 2 (BBN10208) | M | 67 | 25 | n/a | n/a | n/a | n/a | n/a | n/a | n/a | n/a |  |
| CTRL 3 (BBN19653) | F | 55 | 38 | n/a | n/a | n/a | n/a | n/a | n/a | n/a | n/a |  |
| CTRL 4 (NP10/011) | M | 89 | 12 | n/a | n/a | n/a | n/a | II | n/a | n/a | n/a |  |
| CTRL 5 (NP09/136) | M | 85 | 20 | n/a | n/a | n/a | 0 | II | n/a | n/a | n/a |  |
| CTRL 6 (NP13/109) | M | 69 | 34 | n/a | n/a | n/a | n/a | III | n/a | n/a | n/a |  |
|  | **5:1** | **73±5** | **25±4** | **n/a** | **n/a** | **n/a** | **0** | **II** | **2** | **n/a** | **n/a** |  |
| DLB 1 (BBN24369) | F | 90 | 17 | 10 | DLB | n/a | 5 | III | 1 | 18 | n/a |  |
| DLB 2 (BBN24380) | M | 73 | 19 | 12 | DLB | Neocortical | n/a | I | 0 | n/a | n/a |  |
| DLB 3 (BBN003.30937) | M | 82 | 13 | 18 mo | DLB | Neocortical | 6 | I | 1 | n/a | 13 |  |
| DLB 4 (BBN003.32813) | M | 68 | 15 | 17 | DLB | Neocortical | 6 | II | 4 | n/a | n/a |  |
| DLB 5 (BBN003.34162) | F | 79 | 27 | 1 | DLB | Neocortical | 6 | I | 3 | n/a | n/a |  |
| DLB 6 (BBN003.30193) | F | 92 | 16 | n/a | DLB | Neocortical | 5 | II | 5 | n/a | n/a |  |
|  | **3:3** | **81±3.8** | **18±2** | **8.3±15.5** | **DLB** | **Neocortical** | **6** | **I** | **1** | **18** | **13** |  |
| PDD 1 (BBN003.34138) | M | 84 | 15 | 12 | PDD | Neocortical | 6 | III | 2 | n/a | 17 |  |
| PDD 2 (BBN003.35392) | F | 76 | 22 | 23 | PDD | Neocortical | 6 | III | 1 | n/a | n/a |  |
| PDD 3 (BBN003.35795) | F | 82 | 19 | 33 | PDD | Neocortical | 6 | II | 3 | 13 | n/a |  |
| PDD 4 (BBN003.35195) | F | 78 | 21 | 21 | PDD | Neocortical | 6 | III | 3 | 27 | n/a |  |
| PDD 5 (BBN003.28559) | M | 78 | 20 | 20 | PDD | Neocortical | 6 | III | 5 | n/a | n/a |  |
|  | **2:3** | **80±1.5** | **19±1** | **21.8±16.8** | **PDD** | **Neocortical** | **6** | **III** | **3** | **13; 27** | **17** |  |
| **Pontine nucleus analysed: LC** | | | | | | | | | | | |  |
| **Case ID (unique Brain Bank ID)** | **Sex** | **AaD (yrs)** | **PMI** | **DoD (yrs)** | **Neurological dx** | **McKeith LB stage** | **Braak LB stage** | **Braak tau stage** | **Thal phase** | **MMSE score** | **MoCA score** |  |
| CTRL 1 (BBN002.33764) | M | 73 | 19 | n/a | n/a | n/a | 0 | n/a | 2 | n/a | n/a |  |
| CTRL 2 (BBN10208) | M | 67 | 25 | n/a | n/a | n/a | n/a | n/a | n/a | n/a | n/a |  |
| CTRL 3 (BBN21005) | F | 76 | 22 | n/a | n/a | n/a | n/a | n/a | 1 | n/a | n/a |  |
| CTRL 4 (BBN20040) | F | 80 | 22 | n/a | n/a | n/a | n/a | II | n/a | n/a | n/a |  |
| CTRL 5 (BBN13802) | M | 74 | 22 | n/a | n/a | n/a | n/a | II | n/a | n/a | n/a |  |
| CTRL 6 (BBN002.33675) | M | 90 | 12 | n/a | n/a | n/a | 0 | II | n/a | n/a | n/a |  |
|  | **4:2** | **77±3.2** | **20±2** | **n/a** | **n/a** | **n/a** | **0** | **II** | **1; 2** | **n/a** | **n/a** |  |
| DLB 1 (BBN24369) | F | 90 | 17 | 10 | DLB | n/a | 5 | III | n/a | 18 | n/a |  |
| DLB 2 (BBN24380) | M | 73 | 19 | 12 | DLB | Neocortical | n/a | I | 0 | n/a | n/a |  |
| DLB 3 (BBN002.26736) | M | 80 | 46 | 9 mo | DLB | Neocortical | 5 | II | 4 | n/a | n/a |  |
| DLB 4 (BBN002.26330) | M | 66 | 13 | 13 mo | DLB | Neocortical | 3 | n/a | n/a | n/a | n/a |  |
| DLB 5 (BBN002.35813) | F | 81 | 36 | 8 | DLB | Neocortical | 5 | I | n/a | 30 | n/a |  |
| DLB 6 (BBN15706) | M | 72 | 22 | 6 | DLB | Neocortical | 6 | n/a | n/a | 19 | n/a |  |
|  | **4:2** | **77±3.4** | **26±5** | **6.4±11.2** | **DLB** | **Neocortical** |  | **I** | **-** | **18; 19; 30** | **n/a** |  |
| PDD 1 (BBN003.34138) | M | 84 | 15 | 12 | PDD | Neocortical | 6 | III | 2 | n/a | 17 |  |
| PDD 2 (BBN003.35392) | F | 76 | 12 | 23 | PDD | Neocortical | 6 | III | 5 | n/a | n/a |  |
| PDD 3 (BBN003.35795) | F | 82 | 19 | 33 | PDD | Neocortical | 6 | III | 1 | n/a | n/a |  |
| PDD 4 (BBN003.26940) | M | 90 | 27 | 8 | PDD | Neocortical | 6 | II | 3 | n/a | n/a |  |
| PDD 5 (BBN003.28559) | M | 78 | 20 | 20 | PDD | Neocortical | 6 | III | 3 | n/a | n/a |  |
|  | **3:2** | **82±2.4** | **25±7** | **19.2±21.9** | **PDD** | **Neocortical** | **6** | **III** | **3** | **n/a** | **17** |  |

The summary of donor information can be found in a row directly beneath the individual patient donor information for each cohort, providing the male:female ratio, and two measures of central tendency namely the mean ± SEM for AaD, PMI and DoD information and the mode value for the neuropathological and clinical staging data, if such information had been made available to us by the respective Brain Bank. Abbreviations used: AaD: Age at death; CTRL: Control; DLB: Dementia with Lewy Bodies; Duration of disease: DoD; Dx: Diagnosis; F: Female; M: Male; LB: Lewy body; LC: Locus Coeruleus; Mo: Months; N/a: Not available/not applicable; PDD: Parkinson’s disease with Dementia; PPN: Pedunculopontine nucleus; Post-mortem interval: PMI.
