# Supplementary material for: Type‐Specific Single‐Neuron Analysis Reveals Mitochondrial DNA Maintenance Failure Affecting Atrophying Pontine Neurons Differentially in Lewy Body Dementia Syndromes
Source: Aging Cell. 2025 Jun 6;24(8):e70125. doi: 10.1111/acel.70125 (PMC12341794; doi:10.1111/acel.70125)
Supplement: Supplementary file 6 — Table S2. qPCR primers and probes used in the current study. [file ACEL-24-e70125-s004.docx]

**Supplementary Table S2. qPCR primers and probes used in this study.**

| **Primers/Probes** | **Sequence (5'-3')** | **Location** | | **Product size (bp)** | |  |
| --- | --- | --- | --- | --- | --- | --- |
| ND1-F | ACGCCATAAAACTCTTCACCAAAG | m.3458–68 | | 111 | |  |
| ND1-R | GGGTTCATAGTAGAAGAGCGATGG | m.3458–68 | |  |  |  |
| ND1-P | HEX-ACCCGCCACATCTACCATCACCCTC-BHQ1 | m.3492–516 | |  | |  |
| S-ND1-F | CAGCCGCTATTAAAGGTTCG | m.3017–57 | | 1041 | |  |
| S-ND1-R | AGAGTGCGTCATATGTTGTTC | m.3017–57 | |  |  |  |
| ND4-F | ACCTTGGCTATCATCACCCGAT | m.11144–250  m.11144–250 | | 107 | |  |
| ND4-R | AGTGCGATGAGTAGGGGAAGG |  |  |  |  |  |
| ND4-P | FAM-CAACCAGCCAGAACGCCTGAACGCA-BHQ1 | m.11170–94 | |  | |  |
| S-ND4-F | ATCGCTCACACCTCATATCC | m.10534–605  m.10534–605 | | 1072 | |  |
| S-ND4-R | TAGGTCTGTTTGTCGTAGGC |  |  |  |  |  |
| TFAM-F | CCATATTTAAAGCTCAGAACCCAG | 335–456  335–456 | | 122 | |  |
| TFAM-R | CTCCGCCCTATAAGCATCTTG |  |  |  | |  |
| TFAM-P | CY5-TGAATCAGGAAGTTCCCTCCAACGC-BHQ2 | 395–419 | |  | |  |
| S-TFAM-F | CGTTTCTCCGAAGCATGTGG | 143–1279  143–1279 | | 1041 | |  |
| S-TFAM-R | ACAGAACACCGTGGCTTCTA |  |  |  |  |  |
| PINK1-F | GAGTATGGAGCAGTCACTTACAG | 845–988  845–988 | | 144 | |  |
| PINK1-R | CAGCACATCAGGGTAGTCG |  |  |  | |  |
| PINK1-P | ATTO425-CAACTAGCCCCTCACCCCAACAT-BHQ1 | 890–912 | |  | |  |
| S-PINK1-F | GCTTTCGGCTGGAGGAGTAT | 540–1553  540–1553 | | 1014 | |  |
| S-PINK1-R | CCCTCACCAACTGTCTCACG |  |  |  |  |  |
| PGC1α-F | GGAACTGCAGGCCTAACTCC | 1578–715  1578–715 | | 138 | |  |
| PGC1α-R | CTCACTGTACCTGGGCTTCTT |  |  |  |  |  |
| PGC1α-P | FAM-CCACTCCTCCTCATAAAGCCAACCAA-BHQ1 | 1603–28 | |  | |  |
| S-PGC1α-F | GCCGTCTCTACTTAAGAAGCTC | 1130–265  1130–265 | | 1157 | |  |
| S-PGC1α-R | CCCAAGGGTAGCTCAGTTTATC |  |  |  |  |  |
| XPN-F | CGCAATCATAGGACTAGAGACG | 882–1023  882–1023 | | 142 | |  |
| XPN-R | CAGGATGGACTTGTAGGGATG |  |  |  | |  |
| XPN-P | CY5-ATTGATGGTGACCGCATAGACGCC-BHQ2 | | 916–39 | |  | |
| S-XPN-F | CACCGCGAGTAAGGGTGAAT | 98–1103  98–1103 | | 1006 | |  |
| S-XPN-R | CTCACAGCATAGCTGGCCTT |  |  |  |  |  |

The forward (F) and reverse (R) primer sequences used for amplifying the various genes of interest for qPCR analysis and the probe (P) sequences, utilising either a HEX, FAM, CY5 or ATTO425 fluorescent dye attachment for detection and analysis of the P-labelled PCR product, combined with either a BHQ1 or BHQ2 quencher. Custom standard (S) templates were generated using the given sequences to produce a standard curve. Expected product sizes are shown in base pairs (bp). Abbreviations used: bp: Base-pair; F: Forward; P: Probe; R: Reverse; S: Standard.
